# Supplementary material for: An ecological study of the spatiotemporal dynamics and drivers of domestically acquired campylobacteriosis in Ireland, 2011–2018
Source: PLoS One. 2023 Nov 17;18(11):e0291739. doi: 10.1371/journal.pone.0291739 (PMC10655977; doi:10.1371/journal.pone.0291739)
Supplement: S1 Table — (DOCX) [file pone.0291739.s001.docx]

Table S1 Tukey pairwise comparison test results for Clusters and age

| **Clusters** | **Diff.** | **Lower** | **Upper** | **p-value** |
| --- | --- | --- | --- | --- |
| 2-1 | 3.931 | 3.061 | 4.801 | ≤0.001 |
| 3-1 | -0.319 | -2.221 | 1.583 | 0.918^NS^ |
| 3-2 | -4.250 | -6.138 | -2.362 | ≤0.001 |
