# Supplementary material for: An ecological study of the spatiotemporal dynamics and drivers of domestically acquired campylobacteriosis in Ireland, 2011–2018
Source: PLoS One. 2023 Nov 17;18(11):e0291739. doi: 10.1371/journal.pone.0291739 (PMC10655977; doi:10.1371/journal.pone.0291739)
Supplement: S2 Table — (DOCX) [file pone.0291739.s002.docx]

Table S2 Fisher tests results for Clusters and main settlement types (rural, urban, commuter areas)

|  | **Rural** | | | | **Commuter** | | | | **Urban** | | | | |
| --- | --- | --- | --- | --- | --- | --- | --- | --- | --- | --- | --- | --- | --- |
| **Clusters** | **CI 5%** | **aOR** | **CI 95%** | **P-Val** | **CI 5%** | **aOR** | **CI 95%** | **P-Val** | **CI 5%** | **aOR** | **CI 95%** | **P-Val** |  |
| 1 | 1.83 | 1.99 | 2.17 | ≤0.001 | 1.92 | 2.11 | 2.32 | ≤0.001 | 0.38 | 0.41 | 0.44 | ≤0.001 |  |
| 2 | 0.42 | 0.45 | 0.49 | ≤0.001 | 0.33 | 0.36 | 0.40 | ≤0.001 | 2.82 | 3.03 | 3.26 | ≤0.001 |  |
| 3 | 1.32 | 1.57 | 1.87 | ≤0.001 | 2.21 | 2.63 | 3.12 | ≤0.001 | 0.32 | 0.38 | 0.44 | ≤0.001 |  |
