# Supplementary material for: An ecological study of the spatiotemporal dynamics and drivers of domestically acquired campylobacteriosis in Ireland, 2011–2018
Source: PLoS One. 2023 Nov 17;18(11):e0291739. doi: 10.1371/journal.pone.0291739 (PMC10655977; doi:10.1371/journal.pone.0291739)
Supplement: S3 Table — (DOCX) [file pone.0291739.s003.docx]

Table S3 Percentage of total SAs identified as hot/cold spots and statistically unsignificant for each county

| County | % Hotspot | % Non-Significant | % Cold-spot |
| --- | --- | --- | --- |
| Carlow | 8.45 | 89.20 | 2.35 |
| Cavan | 19.20 | 74.61 | 6.19 |
| Clare | 32.09 | 56.75 | 11.15 |
| Cork | 61.07 | 37.01 | 1.92 |
| Donegal | 0.39 | 42.37 | 57.24 |
| Dublin | 31.65 | 66.33 | 2.03 |
| Galway | 41.62 | 49.14 | 9.24 |
| Kerry | 7.97 | 69.27 | 22.76 |
| Kildare | 0.14 | 52.03 | 47.84 |
| Kilkenny | 68.55 | 31.18 | 0.27 |
| Laois | 0.00 | 78.46 | 21.54 |
| Leitrim | 4.05 | 49.71 | 46.24 |
| Limerick | 23.45 | 67.91 | 8.63 |
| Longford | 0.00 | 27.22 | 72.78 |
| Louth | 0.00 | 15.02 | 84.98 |
| Mayo | 3.27 | 84.91 | 11.82 |
| Meath | 0.16 | 25.12 | 74.73 |
| Monaghan | 6.56 | 48.36 | 45.08 |
| Offaly | 4.55 | 88.81 | 6.64 |
| Roscommon | 8.91 | 73.27 | 17.82 |
| Sligo | 0.00 | 88.93 | 11.07 |
| Tipperary | 26.10 | 69.97 | 3.93 |
| Waterford | 67.22 | 32.78 | 0.00 |
| Westmeath | 11.80 | 81.71 | 6.49 |
| Wexford | 26.02 | 73.66 | 0.33 |
| Wicklow | 45.86 | 48.48 | 5.66 |
| Total | 27.14 | 57.50 | 15.36 |
